# Students’ online interaction, self-regulation, and learning engagement in higher education: The importance of social presence to online learning

**DOI:** 10.3389/fpsyg.2022.815220

**Published:** 2022-10-13

**Authors:** Jia Miao, Li Ma

**Affiliations:** ^1^School of Foreign Languages, Tianjin Normal University, Tianjin, China; ^2^School of Management, Yanshan University, Qinhuangdao, China

**Keywords:** online learning, learning engagement, online interaction, self-regulation, social presence

## Abstract

Online learning have played a very significant role for achieving professional and academic qualifications in higher education. There have been more and more researches that explore the issues of learning activities, satisfaction, engagement, and interactions between instructors and students. To promote learning engagement in online learning environments in higher education, this study collected data from 334 full-time undergraduate students in a large public Chinese university and explored the correlation of online interaction, self-regulation learning and social presence on learning engagement in online environments. The research findings indicated that online interaction affected social presence and indirectly affected learning engagement through social presence. In addition, social presence affected learning engagement, self-regulation affected social presence, and social presence also mediated the relationship between self-regulation and learning engagement. This study reported that self-regulation learning and social presence had positive correlation with on students’ learning engagement in online environments. The findings of this study have significant practical implications for teaching practices.

## Introduction

As information and communication technologies have become more widespread in recently years, online learning has been mainstream in higher education, as a significant part of obtaining professional and academic qualifications (Soffer and Nachmias, 2018). There have been more and more researches that explore the issues of learning activities, satisfaction, engagement, and interactions between instructors and students (Allen and Seaman, 2014).

Despite the advantages of online learning, there are still some apprehension and concern as students easily feel disconnected and isolated in online learning environments (Dixson, 2015). So far, disengagement in online learning has been an important predictor of dropout in higher education (Finn and Zimmer, 2012). Developing and maintaining learning engagement can have a significant effect on university or college students’ learning engagement and learning success. Thus, we need design and offer instructional strategies to reduce rate because of disengagement and develop student retention. It is therefore crucial to develop online learning from the perspectives of cognitive, affective, behavioral, and social aspects (Dwivedi et al., 2019). Online learners get distracted easily and get self-regulated difficulty due to the complexity of online learning context (Zimmerman, 2002). Researchers have explored the effectiveness and influence factors of online learning in higher education (Soffer and Cohen, 2019) and found the methods and strategies to improve learning engagement and learning achievement in online environments (Dwivedi et al., 2019).

Prior studies found that a few key factors could affect learning engagement and learning outcomes, such as learning interaction, self-regulation, and social presence (Ng, 2018; Lowenthal and Dunlap, 2020). Students’ success on online learning is affected by their interaction of with their teachers and peers (Moskvicheva et al., 2015; Martin et al., 2018; Guo et al., 2022). Yang et al. (2021) proposed positive relation between teacher–student interaction and teaching presence in online environments. Some studies (Handa, 2020) evidenced positive relationship between teacher–student interaction and learners’ affective engagement. Cheng (2011) examined the role of self-regulation and information commitments on students’ online academic help-seeking. Their research findings indicated enhancing students’ self-regulated learning enables them to utilize sophisticated information commitments in online academic help-seeking activities. The significance of self-regulation on learning achievements in flexible, dynamic, and information-rich types of online learning environments was emphasized (Zheng et al., 2018). Wilson et al. (2018) found that students in an online course who perceived a higher level of social presence placed greater value on greater enjoyment and interest and a lower likelihood of dropping the online course. However, scant attention has been paid to valuable insights on examining the relationship between learning interaction, self-regulation, and social presence and their effects on learning engagement in online environments. Empirical evidence of the influence of online learning interaction, self-regulation, and social presence on online learning engagement has not remained conclusive to date. The study is promising to contribute to have a better understanding about the way of promoting online learning engagement in higher education. This current research can be helpful to open new directions for further studies. To this end, the research questions for the current study are as follows: (1) does online interaction affect social presence and learning engagement in online environments? (2) does self-regulation affect social presence and learning engagement in online environments? (3) does social presence mediate the relationship between self-regulation and learning engagement and between online interaction and learning engagement in online environments?

## Theoretical background hypothesis

### Social presence and learning engagement

A notable characteristic of online learning is a spatial and temporal separation among learners and instructors (Yang et al., 2016). While there is a lack of face to face interaction, such as facial expressions and body language, the learners may feel disconnected or isolated in the social context when communicating in online environments. The ways to support online presence have been explored from different perspectives such as teaching presence and cognitive presence, ensuring instruction and knowledge construction, however, ignoring the social-emotional aspect of online presence. Recent work has paid attention to intersubjective meaning-making in the online teaching and learning context. To this end, more and more researchers have argued that establishing social presence is a key for students to eliminate their emotion of loneliness or isolation in online education. Social presence is defined as the ability of online learners to project themselves socially and emotionally (Garrison, 2007), and can be divided into three aspects, including emotional expression, open communication and group cohesion (Garrison et al., 1999). Social presence has implications of interpersonal connection, belongingness, warmness, and group identification (Rogers and Lea, 2005).

Social presence theory is mostly based on social psychological theories of interpersonal communication and symbolic interaction (Sallnas, 2005). Social presence can increase social, institutional, and academic integration and lead to persistence and online course completion (Tinto, 1987). Online social presence is a complex psychological and social construct in online environments. Online learning is regarded as a whole for promoting students’ learning engagement, which plays a crucial role in leaning performance and achievement. Some researchers declared that learning engagement is not only multifaceted, but also dynamic, context-dependent and interactive (Goldin et al., 2011). Learning engagement is seen as the learners’ interaction with their exterior environment or as the result of learning self-system process (Finn and Zimmer, 2012). It deals with learners’ thought, behavior, and experience toward the learning content (Schindler et al., 2017). While online learners have higher levels of perceived social presence, their learning satisfaction and engagement can be greater degree (Grieve et al., 2016). In sum, the earlier literature offers proof that social presence contributes to promotion of learning engagement. Thus, we propose the following research question:

Hypothesis 1: Social presence positively correlates to learning engagement in online learning environments.

### Online interaction and learning engagement

Online learning offers the chance for online interaction and help learners achieve a response to questions, ideas, and thoughts synchronously and asynchronously. In fact many learners acclaim online learning for presenting a great chance for instructor-learner interaction, learner-learner interaction, and learner-content interaction than the traditional classroom experience (Mahesh and McIssaac, 1999). The literatures have demonstrated that online interaction is important to promote learning performance and learning outcomes in online environments. When learners interact, they are not only more motivated to learn, but also more attentive, participatory and prone to exchange ideas with others (Sims, 2003). For example, online interaction has been shown to support learners’ online learning and academic development (Schneider and Preckel, 2017). In terms of the results of meta-analyses, online learners’ employing collaborative approach in small groups would be more likely to gain better learning performance than their learning individually (Chen et al., 2018). Similarly, learning individually without interaction could lead to learners’ low engagement (Ding et al., 2018). Hara et al. (2000) reviewed previous research about the influences of online interaction and stated that low level learning engagement had been a general phenomenon in higher education. Overall, online interaction was regarded as one of the keys to the success of online education (Picciano, 2002).

Emotional support, as a fundamental aspect of online interaction, has been discussed. Emotional support from instructors and peers through the online interaction can provide the crucial base for online learning. The greater emotional support, the greater will be the effort (Wise et al., 2004). Previous literature has declared that social presence was closely connected with level of interaction, which included instructor-learner interaction, learner-learner interaction, and learner-content interactions. The relationship between social presence and online interaction is positively correlated, which noted that as the level of interaction increases social presence also increases (Tu and McIsaac, 2002). Tu (2000) and Rovai (2002) revealed the relationship between social learning theory and social presence theory in online education and indicated that online interaction was basic to the analysis of this relationship. Trustful, respectful, and intimate relationships among course participants help build a cohesive online learning community in which they feel motivated to support emotional development and knowledge construction. Yukselturk and Yildirim (2008) declared that establishing effective social learning communities could be beneficial to develop higher levels of social presence, which could cause manifested trustful, intimate, and respectful relationship among online learning participants.

Online interaction and social presence are critical elements for encouraging learners’ thinking and learning and motivating them expressing, which developing their engagement in learning environments (Lee et al., 2011; Finn and Zimmer, 2012; Cho and Kim, 2013; Martin and Rimm-Kaufman, 2015). Cho and Kim (2013) stated that online learning with interaction could result in advancing learning engagement among learners. Kim and Lee (2012) proposed that the sense of community and interactivity were connected with learners’ engagement. Accordingly, the following hypotheses are proposed:

Hypothesis 2: Online interaction positively correlates to social presence.

Hypothesis 2a: Online interaction positively correlates to learning engagement.

Hypothesis 2b: Social presence mediates the relationship between online interaction and learning engagement.

### Self-regulation and learning engagement

Another point of online education is online self-regulated learning (Rogers and Swan, 2004). Moreover, self-regulated learning from Zimmerman is seen as one of the theoretical lens of this research. Zimmerman (2002) asserted that self-regulated learning was learners’ capability to consciously and actively controlled and managed their own learning process according to behavior, cognition, and motivation. Dabbagh (2007) stated that a successful online learners should be good at having good interpersonal and communication skills with strong academic self-concept to acquire self-regulated learning strategies.

Previous researches declared self-regulation could reason social presence in a comfortable learning environments. The literature on relationship between self-regulation learning and social presence is limited. For example, Başdoğan (2015) examined two factors of self-evaluation and goal setting had significant association with social presence. While online learner are more self-regulated and more socialized, they are easily engaged in learning. Bolliger and Halupa (2018) explored university students’ self-regulation, transactional distance, perceptions of engagement, and learning outcomes and reported that their relationships were significantly positive. While online learners set learning goals and then regulate their behavior and motivation, they can easily complete communication environment constructing to facilitate emotional expression, open communication and group cohesion among learners in online environments. Tu (2001), and Lai and Hwang (2016) declared that self-regulation, such as goal setting, time management, task strategies, help seeking, and environment structuring, could affect the degree of social presence and eventually could enhance learners’ emotional engagement, behavioral engagement, and learning achievements. Self-regulation and social presence support both academic self-efficacy and learning engagement in gaining educational outcomes (McNamara, 2011). Therefore, based on previous evidence, we propose the following hypothesis in online learning environments:

Hypothesis 3: Self-regulation positively correlates to social presence.

Hypothesis 3a: Self-regulation positively correlates to learning engagement.

Hypothesis 3b: Social presence mediates the relationship between self-regulation and learning engagement.

## Materials and methods

### Participants and procedure

The sample for the current study consisted of 334 full-time undergraduate students (155 males, 179 females, response rate = 90.5%) from a large public Chinese university. The respondents were enrolled in a variety of 1–4 year undergraduate courses, but majority respondents were second (40.7%) and third year (44.9%) undergraduates. All participants had experienced at least an online course prior to this survey. Study-discipline-wise, 15.6% were studying history, 14.9% were in management courses, 16.6% were in physics courses, 13.2% were doing their engineering, 15.1% belonged to computer courses, 17.6% were studying administration, and the rest belonged to the “others” category. Data were collected by distributing paper-and-pencil questionnaires in the classrooms.

### Measures

All scales in this study used a 5-point scale ranging from 1 for “strongly disagree” to 5 for “strongly agree.” Online learning engagement scale (OLE) was adopted and modified from Sun and Rueda (2012). The scale has three dimensions: behavioral engagement (three items), emotional engagement (six items), and cognitive engagement (five items). Sample items were, “I complete videos and exercises on time” and “I learn the online course even when there are no quizzes that week.” The coefficient alpha reliability for this scale was 0.905.

Social presence was measured from Online Social presence questionnaire (OSPQ) by Eunmo and Richard (2012). There are 5 dimensions in this scale: social respect (five items), social sharing (five items), open mind (three items), social identity (four items), and intimacy (two items). The sample items were “I feel a sense of presence when students and the instructors have a variety characteristic in my online community” and “I feel a sense of presence when students and instructor call me by my name.” The coefficient alpha reliability was 0.909.

Self-regulation was measured from Online Self-Regulated Learning Questionnaire (OSLQ) by Barnard et al. (2009). This scale has 6 dimensions: goal setting (five items), environment structuring (four items), task strategies (four items), time management (three items), help-seeking (four items) and self-evaluation (three items). Example items include, “I set standards for my assignments in online courses” and “I summarize my learning in online courses to examine my understanding of what I have learned.” The coefficient alpha for this scale was 0.925.

Online interaction was measured with a scale with eighteen items from the previous research regarding student interaction and satisfaction in online education (Kuo et al., 2014). Three dimensions included in this scale: learner-learner interactions (eight items), learner-instructor interactions (six items), and learner-content interactions (four items). Example items include, “I got lots of feedback from my classmates” and “Online course materials stimulated my interest for this course.” The coefficient alpha reliability for this scale was 0.897.

#### Control variables

We used the following control variables: gender, grade, learning duration (every day), and these variables were chosen based on prior research studies learning engagement.

## Results

### Descriptive statistics

The means, standard deviations, correlations, and reliabilities for all variables used in the present study are presented in Table 1. The online learning engagement was positively related to social presence (r = 0.693, *p* < 0.01); self-regulation (r = 0.715, *p* < 0.01) and online interaction (r = 0.605, *p* < 0.01). Moreover, social presence was positively related to self-regulation (r = 0.671, *p* < 0.01) and online interaction (r = 0.632, *p* < 0.01), while self-regulation was positively related to online interaction (r = 0.721, *p* < 0.01).

**TABLE 1:** Descriptive statistics, correlation, and reliabilities for all variables.

Variables	M	SD	1	2	3	4	5	6	7
1. Gender	0.66	0.476	NA						
2. Grade	2.43	0.808	0.015	NA					
3. LL	2.54	1.156	0.027	0.069	NA				
4. LE	3.327	0.686	–0.070	0.134*	0.223**	0.905			
5. SP	3.256	0.752	–0.034	0.052	0.169**	0.693**	0.909		
6. SR	3.366	0.588	–0.093	0.170**	0.196**	0.715**	0.671**	0.925	
7. OI	3.460	0.587	–0.054	0.136*	0.115*	0.605**	0.632**	0.721**	0.897

LD, learning duration; LE, learning engagement; SP, social presence; SR, self-regulation; OI, online interaction; Cronbach’s α is in italics on the diagonal (*N* = 334).

*p < 0.05, **p < 0.01.

### Confirmatory factor analysis

Confirmatory factor analysis (CFA) was used to examine the discriminant validity of the four main variables in this study (i.e., learning engagement; social presence; self-regulation; online interaction). Considering the relatively small sample size, we created parcels of the measured variables before conducting CFA following the content-based parceling method recommended by Landis et al. (2000). When there were a larger number of dimensions within a scale using this content-based method, each dimension was parceled into one item. Therefore, LE was parceled into three items, SP was parceled into five items, OI was parceled into three items and SR was parceled into six items. The CFA results were presented in Table 2. As Table 2 presents, the hypothesized four-factor model (χ2 = 361.356, *df* = 113, RMSEA = 0.08, CFI = 0.929, NFI = 0.901, TLI = 0.915, IFI = 0.930) met these suggested criteria and was better than other alternative models. These results mean that there is a good discriminant validity among the four key variables used in the present study.

**TABLE 2:** Measure model comparison.

Models	χ2	*df*	RMSEA	CFI	NFI	TLI	IFI
Four-factor model (LE; SP; SR; OI)	361.356	113	0.79	0.929	0.901	0.915	0.930
There-factor model A (LE; SR; OI + SP)	543.721	116	0.105	0.878	0.851	0.857	0.879
There-factor model B (LE; SP + SR; OI)	660.907	116	0.119	0.845	0.819	0.818	0.846
Two-factor model (LE + SR; OI + SP)	597.085	118	0.108	0.864	0.837	0.843	0.865
One factor model (LE + SP + SR + OI)	742.057	119	0.125	0.823	0.797	0.798	0.824

LE, learning engagement; SP, social presence; SR, self-regulation; OI, online interaction; OI + SP, online interaction and social presence were combined into one factor.

### Testing the hypothesized model

**FIGURE 1:**
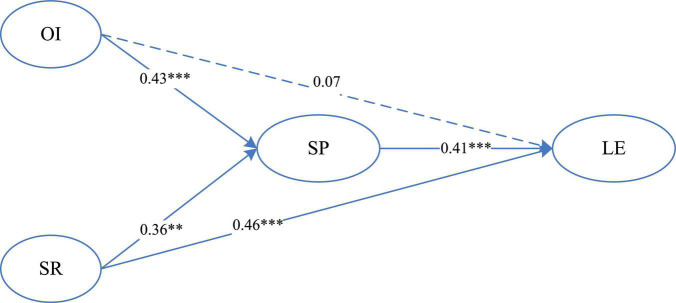
Structural model of the associations among self-regulation and online interaction, social presence and learning engagement. **p < 0.01, ***p < 0.001 (χ^2^ = 377.86, *df* = 127, CFI = 0.93, IFI = 0.93, TLI = 0.92, NFI = 0.90, RMSEA = 0.077).

Structural Equation Modeling (SEM) was used to test hypotheses in this study. The entire data analysis was conducted using the IBM AMOS 24.0 software package. The results were presented in Figure 1.

All the path coefficients presented in the model (Figure 1) were significant at the 0.01 level, except for the path coefficient between online interaction and learning engagement. The goodness-of-fit indices for this model (CFI = 0.93, IFI = 0.93, TLI = 0.92, NFI = 0.90, RMSEA = 0.077) indicated that the hypothesized model fit the sample data well. Results showed that social presence significantly predicted learning engagement (β = 0.41, *p* < 0.001), while, online interaction (β = 0.43, *p* < 0.001), and self-regulation (β = 0.36, *p* < 0.01) significantly predicted social presence. Consequently, hypothesis 1, hypothesis 2, and hypothesis 3 were supported. The path coefficient between online interaction and learning engagement (β = 0.07) was not significant which mean hypothesis 2a was not supported. Next, self-regulation (β = 0.46, *p* < 0.001) significantly predicted learning engagement. So hypothesis 3a was supported.

Finally, the bootstrapping and an estimated bias-corrected 95% confidence interval were used to test the significance of the above mediating effects according to Preacher and Hayes (2008). Results in Table 3 showed that, the indirect effect (95% confidence interval) on the association between online interaction and learning engagement was [0.177, *p* < 0.01, 95% CI: (0.047, 0.437)], and the indirect effect (95% confidence interval) on the association between self-regulation and learning engagement was [0.147, *p* < 0.05, 95% CI: (0.018, 0.336)]. Table 3 showed that the two 95% confidence intervals did not include zero, which indicated the significant mediating role of social presence in both associations. Therefore, hypothesis 2b and hypothesis 3b were supported. Taken together, the results indicated that social presence partially mediated the relation between online interaction and learning engagement, and completely mediated the relation between self-regulation and learning engagement.

**TABLE 3:** Path coefficients of the model.

Path	Indirect effect	SE	95% CI		*p*
			Lower	Upper	
OI—SP—LE	0.177	0.098	0.047	0.437	<0.01
SR—SP—LE	0.147	0.085	0.018	0.336	<0.01

## Discussion

The results of this study indicate that online interaction, self-regulation, and social presence play significant roles in predicting students’ learning engagement. Unexpectedly, online interaction did not directly influence learners’ learning engagement. It indirectly affected learning engagement through online social presence. As a result, it is necessary to integrate interactions, self-regulation, and social presence to promote university students’ learning engagement in online environments. The findings of the current study can provide academic and practical insights to help instructors design online courses effectively in higher education.

The role of social presence in establishing a trusting climate was confirmed by Grieve et al. (2016), who examined the social presence and its relations to perceived learning. The study reported that social presence contributed to perceived learning more as a socio-emotional source, while leaving cognitive source unaffected. Richardson and Swan (2003) found that learners could project themselves emotionally and socially in learning activities involving online interaction, to this end social presence played a crucial role in advancing students’ satisfaction.

Online interaction did not directly influence learning engagement. Leong (2011) explained that if online learners focused on only interactive behavior and ignored emotional expression and open communication with instructors or peers, it was difficult for them to achieve learning behavioral engagement, emotional engagement, and cognitive engagement. Moreover, designed interaction refers to having a high cooperative or collaborative one. As indicated direct impacts of interaction on online learning engagement were not observed in this study. Nonetheless, it indirectly affected learning engagement through social presence (Cho and Kim, 2013). This result is quite similar to the subjective perspective of Picciano (2002) study, which found that social presence had an influence on learning engagement due to online interaction. Moreover, the results found that a similar concept of subjective output that was proposed in Cho and Kim’s (2013) study. However, the results from Tolmie et al.’s (2010) study supported a different concept of learning output. The reason for this might be that interaction learning provided learners with social communication and information sharing, such as argumentation and negotiation, cognitive elaboration, and the social construction of knowledge in online environments from the respective of cognitive online learning. Some earlier literature on online higher-education concluded that social presence impacted learning outcomes via online learning strategy. Similarly, the mediation tests in the current study showed in detail how social presence mediated the relationship between online interaction and the focal learning outcomes and therefore revealed the operation of an affective, motivational, and behavioral learning process during the transformation of perceptions of learning into learning outcomes in online environments.

This study also focuses on investigating the relationships among students’ self-regulation, social presence, and learning engagement in online environments with the theoretical framework of self-regulation theory, social presence theory, and engagement theory. Christopherson (2011) classified interaction and social presence as external factor of affecting online learning and self-regulation as internal factor. The modeling in this current study identified an effect size of online self-regulation learning on social presence, which agreed with some quasi-experimental studies’ findings that learners’ self-regulation learning strategy could be improved through structured and intentional learning or teaching practice. For example, Jansen et al. (2019) stated that an online pedagogical intervention incorporating self-regulation training in online environments was efficient at promoting students’ learning engagement. This study examined online self-regulation’s influence on social presence and learning engagement and accepts statements in earlier research. Considering online self-regulation (e.g., task analysis, self-observation, self-reaction, self-control, and self-motivational beliefs) favors fostering open communication and group cohesion of social presence and promoting learning self-efficacy in online environments. In terms of self-determination theory, learners may be inclined to be motivated and engaged in online learning when self-regulation, social presence, and self-efficacy psychological needs are met (Ryan and Deci, 2000; Lo and Hew, 2020).

## Limitations and further directions

This study has some limitations. First, due to the cross sectional approach of the data collection, the causal relationships should be interpreted with caution. Second, this study was limited to examining the effect of self-regulation, online interaction, and social presence on student engagement, not learning outcomes or achievement. Third, this study collected and analyzed quantitative data from only a questionnaire, ignoring other forms for collecting data.

Future research could further validate the model in terms of longitudinal studies dealing with surveys as well as other procedures (e.g., observation and interview). Furthermore, future researchers need focus on the relationship between self-regulation, online interaction, social presence, and learning engagement and explore students’ learning outcomes, such as grades and learning gains. Finally, it is strongly recommended to use diverse data collection methods, such as mixed-methods (e.g., interviews, focus group interviews, observations, reflecting log etc.) for more meaningful research findings.

## Conclusion and implications

This study is conducted to explore the effects of self-regulation, interaction, and social presence on learning engagement in online environments in higher education in China. The results confirms that social presence directly affects learning engagement, self-regulation directly affects learning engagement, and social presence mediates the relationship between online interaction and learning engagement and between self-regulation and learning engagement. The findings of this present research have significant practical implications for teaching practices in Chinese higher education.

First, this study emphasizes the significance of online self-regulated learning when learners interact and promotes online learning and theory of community of inquiry. Enhancing the sense of social presence is now regarded as an important mechanism for fostering learning engagement and active learning. Second, instructors need be aware of learners’ negative emotion, such as loneliness, disconnectedness, and boredom, since emotional engagement and sense of social presence may be reduced. Third, considering strategies for developing social presence is very important and essential, since online learning has the isolated nature. Teachers should conduct research on social presence measurement and its effectiveness and on formulating principles of designing social presence in higher education. Finally, online instructors are expected to design online interacting activities and develop learning materials to maximize learning engagement and learning outcomes.

## Data availability statement

The original contributions presented in this study are included in the article/supplementary material, further inquiries can be directed to the corresponding author.

## Ethics statement

The studies involving human participants were reviewed and approved by Northeastern University Ethics Committee. The participants provided their written informed consent to participate in their study. Written informed consent was obtained from the individuals for the publication of any potentially identifiable images or data included in this article.

## Author contributions

JM designed the study, performed the research, analyzed the data, and wrote the manuscript. LM developed the idea for the study, carried out additional analyses, and finalized the manuscript. Both authors contributed to the article and approved the submitted version.
